# Solid-solution alloy nanoparticles of a combination of immiscible Au and Ru with a large gap of reduction potential and their enhanced oxygen evolution reaction performance†
†Electronic supplementary information (ESI) available. See DOI: 10.1039/c9sc00496c


**DOI:** 10.1039/c9sc00496c

**Published:** 2019-04-25

**Authors:** Quan Zhang, Kohei Kusada, Dongshuang Wu, Naoki Ogiwara, Tomokazu Yamamoto, Takaaki Toriyama, Syo Matsumura, Shogo Kawaguchi, Yoshiki Kubota, Tetsuo Honma, Hiroshi Kitagawa

**Affiliations:** a Division of Chemistry , Graduate School of Science , Kyoto University , Kitashirakawa- Oiwakecho, Sakyo-ku , Kyoto 606-8502 , Japan . Email: kusada@kuchem.kyoto-u.ac.jp ; Email: kitagawa@kuchem.kyoto-u.ac.jp; b Department of Applied Quantum Physics and Nuclear Engineering , Kyushu University , 744 Motooka, Nishi-ku , Fukuoka 819-0395 , Japan; c The Ultramicroscopy Research Center , Kyushu University , Motooka 744, Nishi-ku , Fukuoka 819-0395 , Japan; d INAMORI Frontier Research Center , Kyushu University , Motooka 744, Nishi-ku , Fukuoka 819-0395 , Japan; e Japan Synchrotron Radiation Research Insitute (JASRI) , SPring-8, 1-1-1 Kouto, Sayo-cho, Sayo-gun , Hyogo 679-5198 , Japan; f Department of Physical Science , Graduate School of Science , Osaka Prefecture University , 1-1 Gakuen-cho, Naka-ku, Sakai , Osaka 599-8531 , Japan

## Abstract

Au and Ru are elements that are immiscible in the bulk state and have the largest gap in reduction potential among noble metals. Here, for the first time, Au_*x*_Ru_1–*x*_ solid-solution alloy nanoparticles (NPs) were successfully synthesized over the whole composition range through a chemical reduction method. Powder X-ray diffraction and scanning transmission electron microscopy coupled with energy-dispersive X-ray spectroscopy showed that Au and Ru atoms are homogeneously mixed at the atomic level. We investigated the catalytic performance of Au_*x*_Ru_1–*x*_ NPs for the oxygen evolution reaction, for which Ru is well known to be one of the best monometallic catalysts, and we found that even alloying with a small amount of Au could significantly enhance the catalytic performance.

## Abstract 2

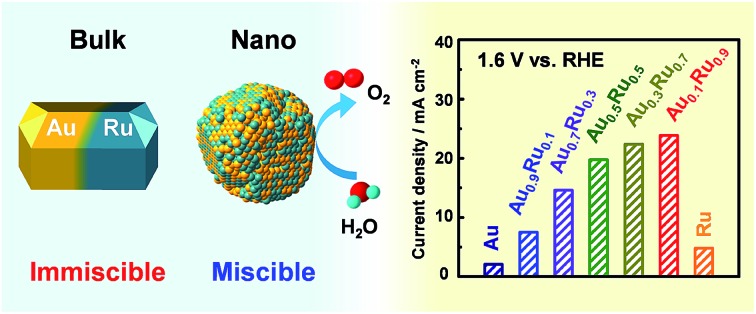
Au*_x_*Ru_1–*x*_ with whole compositions were synthesized and showed an enhanced catalytic performance for OER.

## Introduction

Noble metals (Ru, Rh, Pd, Ag, Os, Ir, Pt, and Au) show their own unique properties and excellent performance as catalysts,^1^–^4^ despite being minor elements and expensive. They are used as nanoparticles (NPs) for industrial applications; for example, Ru is well known for ammonia synthesis,^5^–^7^ Rh is used for NO_*x*_ purification of car exhaust,^8^,^9^ and Au is used as an oxidative esterification catalyst.^10^–^13^ On the other hand, noble metal alloy NPs have received much attention during the past few decades, because they show synergetic properties derived from each constituent metal.^14^–^19^ Among noble metal alloy NPs, solid-solution alloy NPs, in which different metals are randomly mixed at the atomic level, are more promising than other types of alloys such as the core–shell or segregated type to continuously tune the properties because their electronic states can be continuously controlled by changing compositions.^20^ However, the development of solid-solution alloy NPs has long been limited by the immiscibility of the constituents, because most of the metal combinations are immiscible in the bulk state.^21^ Although several new solid-solution alloy NPs in immiscible systems such as Ag–Rh^22^ and Pd–Ru^23^ have been recently obtained, it is still challenging to synthesize solid-solution alloy NPs, particularly those that have a large gap of reduction potential between the constituent metals.

The key point in the synthesis of these solid-solution noble alloy NPs is the concurrent reduction of metal precursors.^24^,^25^ If the two kinds of metal ions are not reduced simultaneously, phase-separated NPs, such as core–shell or segregated types, would be obtained.^26^ However, it is not easy to simultaneously reduce two kinds of noble metal ions that have a large gap of reduction potential causing a remarkable difference in reduction speed, such as Au and Ru which have the largest gap among noble metals (Ru^3+^ + 3e^–^ ⇌ Ru, *E*° = 0.455 V *vs.* Au^3+^ + 3e^–^ ⇌ Au, *E*° = 1.498 V, as shown in Table S1† 27).^24^,^25^

Ru is one of the best known highly active catalysts for the oxygen evolution reaction (OER).^28^,^29^ However, Ru catalysts are generally unstable in acidic solutions because Ru is easily oxidized in the working potential range of the OER.^30^–^32^ Au is known as the most stable metal and is used to improve the stability of metal catalysts.^33^–^35^ Actually, it is recently reported that even phase separated Au–Ru NPs show an enhanced OER performance.^36^ Therefore, the alloying of Au and Ru at the atomic level could be an effective way to improve the catalytic performance of Ru OER catalysts. In general, varying the composition of the solid-solution alloy has a great significance on its properties because the electronic structure of the alloy can be changed with its composition.^22^,^23^,^37^ Thus, it is interesting and challenging to create Au_*x*_Ru_1–*x*_ solid-solution alloy NPs over the entire composition range and to further systematically investigate their OER catalytic performance.

In this study, we carefully chose metal precursors with suitable ligands to overcome the limitation of the reduction potential difference and successfully obtained AuRu solid-solution alloy NPs over the entire composition range for the first time, although Au and Ru are immiscible throughout the entire composition range in the bulk state even at high temperatures up to their melting points (Fig. S1†).^38^ We also examined the OER catalytic performance of Au_*x*_Ru_1–*x*_ NPs and found that the activity continuously changed with composition and the alloy NPs exhibited an enhanced performance compared with pure Au and Ru.

## Results and discussion

### Synthesis and characterization

To synthesize the Au_*x*_Ru_1–*x*_ solid-solution NPs, we controlled the reduction speed of the precursors by choosing appropriate coordinating ligands of the metal precursors because the ligands can profoundly affect the ions' stability and reduction potential, and thus their reduction kinetics can be desirably tuned.^25^ Potassium pentachloronitrosylruthenate(ii) (K_2_Ru(NO)Cl_5_)^39^ and hydrogen tetrabromoaurate(iii) hydrate (HAuBr_4_·*n*H_2_O) were chosen as metal precursors to form the solid-solution alloy NPs. Au_*x*_Ru_1–*x*_ solid-solution NPs were synthesized using a polyol reduction method. First, the precursors were dissolved in 10 ml diethylene glycol (DEG) with an appropriate molar ratio. Then, the metal precursor solution was slowly dropped into 100 ml ethylene glycol (EG) solution containing 444 mg poly(vinylpyrrolidone) (PVP) at 190 °C. The temperature of the solution was maintained at 190 °C during the dropping process. The NPs were separated by centrifugation after cooling to room temperature. The details of the experimental conditions are described in the ESI.†

The prepared samples were characterized by transmission electron microscopy (TEM) (Fig. S2†). The mean diameters of the Au_*x*_Ru_1–*x*_ (*x* = 0.1, 0.3, 0.5, 0.7, and 0.9) NPs were determined from the TEM images to be 6.6 ± 1.4, 15.7 ± 2.9, 15.4 ± 2.5, 15.3 ± 2.3, and 15.3 ± 2.7 nm, respectively. The particle size of Au_0.1_Ru_0.9_ is relatively smaller compared to other compositions. This may be related to the nature of Ru. X-ray fluorescence (XRF) and energy-dispersive X-ray (EDX) analyses confirmed the atomic ratios of Au and Ru in the prepared NPs (Table S2†). These results are consistent with the nominal ratios of the metal precursors used in the synthesis.

**Fig. 1:**
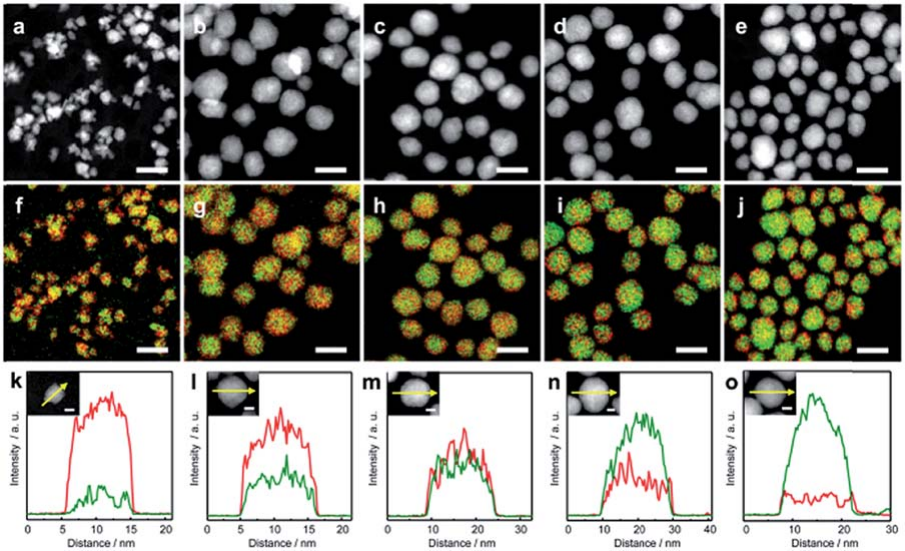
High-angle annular dark-field scanning transmission electron microscopy (HAADF-STEM) images of (a) Au_0.1_Ru_0.9_, (b) Au_0.3_Ru_0.7_, (c) Au_0.5_Ru_0.5_, (d) Au_0.7_Ru_0.3_, and (e) Au_0.9_Ru_0.1_ NPs. (f)–(j) are the corresponding overlay images of the Ru-L and Au-L STEM energy-dispersive X-ray (EDX) maps of (a)–(e). (k)–(o) are the EDX line profiles of the NPs along the arrows shown in the inset figures. Au and Ru are indicated in green and red colors, respectively. The scale bars shown in (a)–(j) and the inset figures of (k)–(o) are 20 and 5 nm, respectively.

To clarify the structure of the obtained AuRu NPs, high-angle annular dark-field scanning TEM (HAADF-STEM) and EDX elemental mapping of Au and Ru were carried out. HAADF-STEM images and the maps of Au and Ru elemental distribution on the obtained Au_*x*_Ru_1–*x*_ NPs are shown in Fig. 1a–e and f–j, respectively. Au-L and Ru-L STEM-EDX maps of Au_*x*_Ru_1–*x*_ NPs are separately shown in Fig. S3.† These results give direct evidence of the homogeneous distribution of Au and Ru atoms in each particle. The typical EDX line scan profiles shown in Fig. 1k–o also demonstrate that Au and Ru atoms are well distributed in the particles, and the metal composition of the particles gradually changes from Ru-rich to Au-rich. These results show the formation of Au_*x*_Ru_1–*x*_ solid-solution alloy NPs over the entire composition range.

**Fig. 2:**
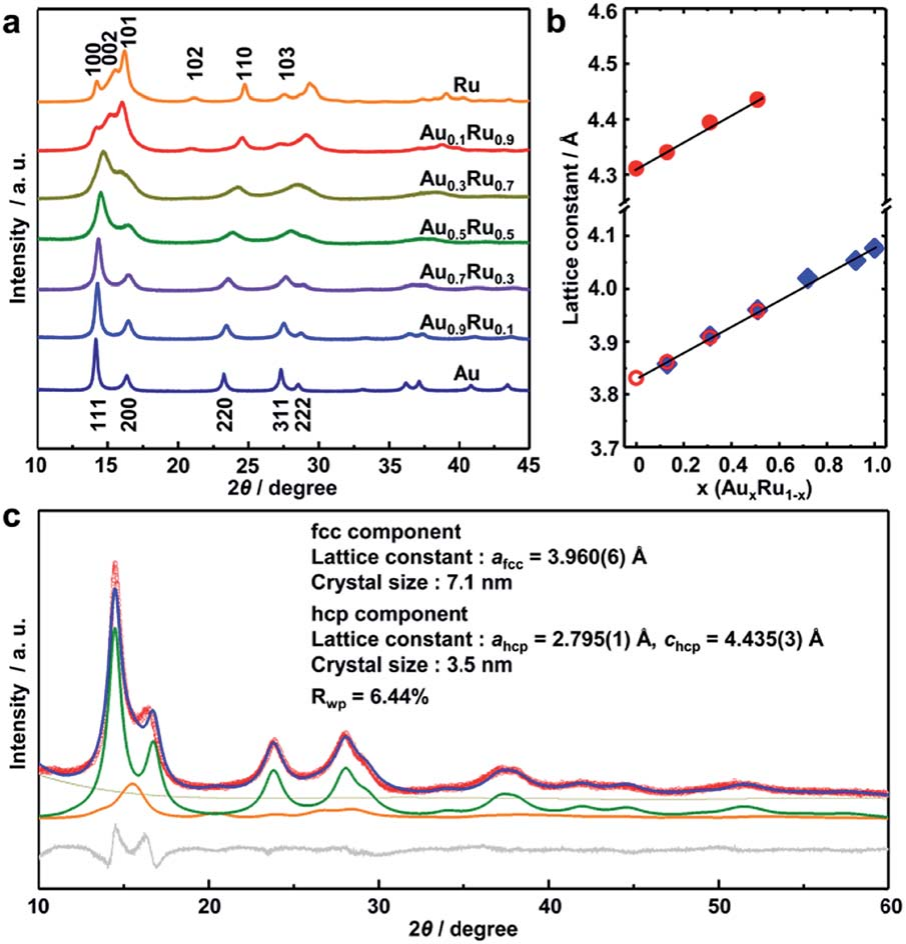
(a) The synchrotron powder XRD patterns (2*θ* = 10–45°) of Au_*x*_Ru_1–*x*_ NPs at 303 K. The radiation wavelength was 0.58068(1) Å. (b) Dependence of the lattice constant on the metal composition in Au_*x*_Ru_1–*x*_ NPs. ○(red), [black circle](red), and ♦(blue) indicate the lattice constant *a*_hcp_ and *c*_hcp_ of the hcp component and *a*_fcc_ of the fcc component, respectively. (c) The diffraction pattern of Au_0.5_Ru_0.5_ NPs (red circles) at 303 K and the calculated profile (blue line) by Rietveld refinement. The profiles of the difference, background, and the fcc and hcp components are shown as gray, dark yellow, green and orange lines, respectively.

The crystal structure of the obtained AuRu NPs was investigated by synchrotron powder X-ray diffraction (XRD) analysis at BL02B2, SPring-8.^40^ The XRD patterns of Au_*x*_Ru_1–*x*_ NPs gradually change from face-centered cubic (fcc) to hexagonal closed-packed (hcp) patterns with increasing amounts of Ru (Fig. 2a). We then performed Rietveld refinement on each pattern of the alloy NPs (Fig. 2b, c and S4–S7†). The best fit of Au_0.5_Ru_0.5_ was obtained with two components of fcc and hcp (Fig. 2c). The lattice constant of the fcc component was calculated to be 3.960(6) Å, which is smaller than that of Au NPs (*a* = 4.077(1) Å, Fig. S8†). For the hcp component, the lattice constants were 2.795(1) and 4.435(3) Å for *a*_hcp_ and *c*_hcp_, which were larger than those of Ru NPs (*a* = 2.709(5) and *c* = 4.307(8) Å, Fig. S9†). As both hcp and fcc are close-packed structures, the lattice parameter *a*_fcc_ in a fcc structure is nearly √2*a*_hcp_ in an hcp structure. Given that the lattice constant follows Vegard's law,^41^ the Au : Ru compositions of the fcc and the hcp phases were estimated to be 0.52 : 0.48 and 0.50 : 0.50, which are similar to the results of EDX and XRF analyses. These results strongly suggest that Au_0.5_Ru_0.5_ NPs contain two phases; however, both phases are solid-solution structures with the same composition. The lattice constants of Au_*x*_Ru_1–*x*_ NPs increased linearly with increasing Au content (*x*) as estimated by Rietveld refinement (Fig. 2b). The linear correlation between the composition and the lattice constant follows Vegard's law, which also confirmed the formation of the solid-solution AuRu alloy over the entire composition range.

### Catalytic properties

The electrocatalytic OER activity of Au_*x*_Ru_1–*x*_ NPs was investigated in a 0.05 M H_2_SO_4_ solution in a standard three-electrode system with a Pt wire and an Ag/AgCl (3.5 M NaCl) electrode as the counter and reference electrodes, respectively. The synthesized NPs were first loaded on carbon black (VXC 72R) with 20 wt% metal (Fig. S10†). The catalysts were uniformly cast onto a rotating disk electrode for recording *iR*-corrected OER polarization curves at a scan rate of 5 mV s^–1^. The working electrode was continuously rotated at 1600 rpm during the measurements. The Au and Ru catalysts were also measured as a reference.

**Fig. 3:**
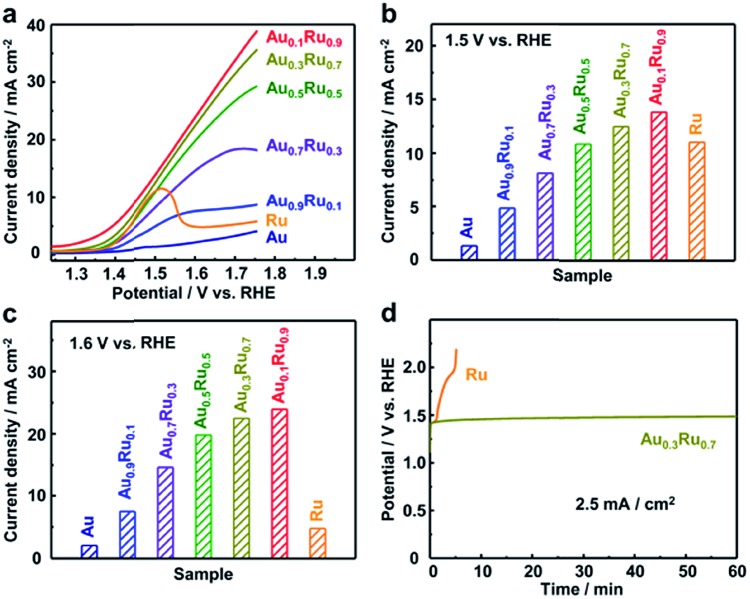
(a) Linear sweep voltammetry (LSV) polarization curves of the OER catalyzed by Au_*x*_Ru_1–*x*_ catalysts. The current density of each catalyst at potentials of (b) 1.5 V and (c) 1.6 V. (d) Chronopotentiometry curves of the Au_0.3_Ru_0.7_ catalyst compared to the Ru catalyst at a constant current density of 2.5 mA cm^–2^ for 1 h. All the tests were performed in an Ar-saturated 0.05 M H_2_SO_4_ solution at a scan rate of 5 mV s^–1^. All the polarization curves were collected with *iR*-correction.

Fig. 3a shows the linear sweep voltammetry (LSV) curves of the Au_*x*_Ru_1–*x*_ catalysts. The current densities of each catalyst at potentials of 1.5 and 1.6 V are shown in Fig. 3b and c. For the Ru catalyst, the current density has the highest value at around 1.5 V, but gradually reduces after that, which is caused by the dissolution of Ru with the potential increasing.^30^–^32^ The Au catalyst does not show obvious catalytic activity.^42^ The Au_*x*_Ru_1–*x*_ catalysts demonstrated the composition dependence of the catalytic performance. With increasing Ru content, the current density becomes higher. More importantly, Au is inactive for the OER; nevertheless, Au_0.3_Ru_0.7_ and Au_0.1_Ru_0.9_ catalysts show higher activity than Ru at 1.5 V. The current densities of the Au_*x*_Ru_1–*x*_ catalysts continuously increase with the potential increasing and are higher than that of the Ru catalyst at 1.6 V. This confirms the stability improvement of the Au_*x*_Ru_1–*x*_ catalysts. We further investigated the stability of Au_0.3_Ru_0.7_ and Au_0.1_Ru_0.9_ catalysts by chronopotentiometry tests at a constant current density of 2.5 mA cm^–2^ for 1 h (Fig. 3d and S11†).^43^,^44^ The curves show the potential change at a current density of 2.5 mA cm^–2^. The potential of the Ru catalyst quickly changed from 1.4 to above 2.0 V in 5 min, essentially losing all its activity. In contrast, the Au_0.3_Ru_0.7_ and Au_0.1_Ru_0.9_ catalysts showed a much slower deactivation during the stability test. From these results, it can be concluded that the alloy catalysts show much higher stability than the pure Ru catalyst.

**Fig. 4:**
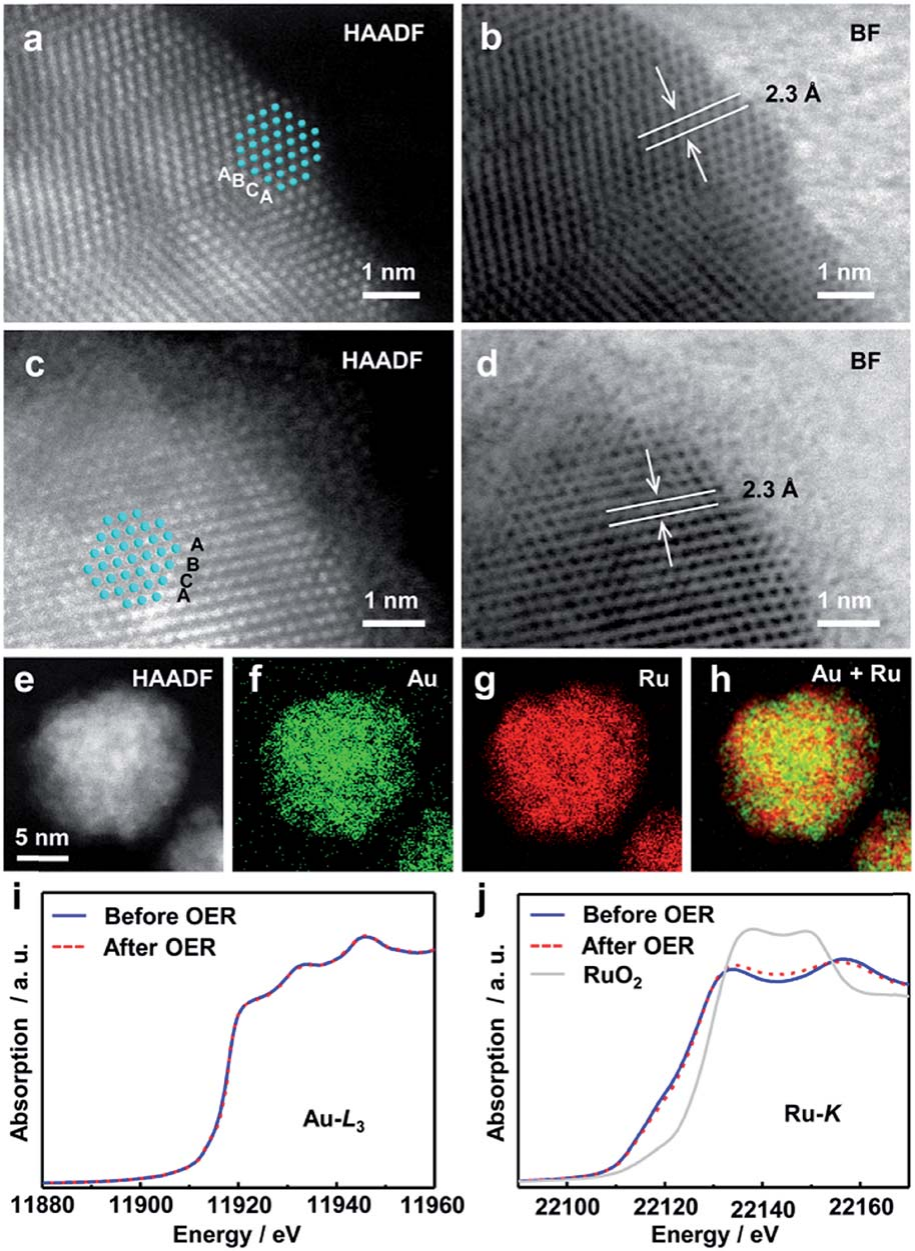
HAADF-STEM images of the Au_0.3_Ru_0.7_ catalyst before (a) and after (c) the chronopotentiometric stability measurement. (b) and (d) BF images of the NPs in (a) and (c). (e) HAADF-STEM image of the Au_0.3_Ru_0.7_ catalyst after the stability measurement, (f)–(h) Au-L (green), Ru-L (red), overlay (Ru + Au) STEM-EDX maps of NPs in (e). Au L_3_-edge (i) and Ru K-edge (j) XANES spectra of the Au_0.3_Ru_0.7_ catalyst before and after OER stability measurement.

We then investigated the structures of the Ru, Au_0.3_Ru_0.7_ and Au_0.1_Ru_0.9_ catalysts after the stability test by TEM, STEM-EDX and X-ray absorption near-edge structure (XANES). From TEM observations, we found that there were no Ru NPs on the carbon in the Ru/C catalyst after the test due to the rapid oxidation and dissolution of Ru (Fig. S12a†).^30^–^32^ In contrast, a large amount of Au_0.3_Ru_0.7_ or Au_0.1_Ru_0.9_ NPs remained on the carbon support (Fig. S12b and c†). We further analyze the structure of the alloy catalysts after the OER with Au_0.3_Ru_0.7_. HAADF- and bright field (BF)-STEM images (Fig. 4a–d) show the comparison of the alloy structures before and after the stability test. Both of the particles show the same fcc lattice and {111} interplanar spacing, indicating that the metallic structure of alloy NPs was mostly retained. However, a thin amorphous layer was observed on the alloy surface after the stability test. STEM-EDX analysis confirmed the good distributions of Au and Ru in the NPs (Fig. 4e–h, S13 and S14†). We further investigated the structure of the catalysts with XANES. The Au L_3_-edge and Ru K-edge spectra confirmed that the original alloy structure was maintained after the stability test, and indicated that the thin amorphous layer on the alloy surface would be RuO_*x*_ (Fig. 4i–j).

To elucidate the enhancement of the catalytic performance, the change in the electronic structure caused by atomic level alloying of Au and Ru was investigated by XPS (Fig. S14, Table S3†). The Au 4f peaks of the alloys shifted to higher energy with increasing Ru content. By contrast, with the Au content increasing, the Ru 3p peaks of the alloys shifted to a lower energy. These results indicate electron transfer from Au to Ru in alloy NPs. According to the mechanism of the OER in an acid solution (Table S4†), the formation of intermediate oxygen species on the surface of the catalyst (*e.g.*, *OH, *O, and *OOH; * represent active sites on the metal surface) is a key step for the OER process.^45^ These steps would be significantly affected by the change in the electronic structure of the catalyst. Thus, the change of the electronic structure in Au_*x*_Ru_1–*x*_ NPs could lead to better balance between adsorption and dissociation energies for oxygen species and further enhance the catalytic activity.^46^,^47^ At the same time, the electron transfer from Au to Ru in Au_*x*_Ru_1–*x*_ NPs could suppress the oxidation of Ru, which would be one of the origins of the enhancement in the stability of the Au_*x*_Ru_1–*x*_ alloy catalysts. Therefore, the alloying of Ru with Au improves the activity and stability of Ru as the OER catalyst in acid solutions.

## Conclusions

In summary, we synthesized and characterized Au_*x*_Ru_1–*x*_ solid-solution NPs over the whole composition range for the first time, where Au and Ru have the largest gap in reduction potential among noble metals and are completely immiscible even at high temperatures up to their melting points in the bulk state. STEM-EDX and PXRD results demonstrated that Au and Ru were randomly and homogeneously distributed in a single NP. The alloy catalysts showed an enhanced catalytic performance compared with Ru which is known as one of the best monometallic OER catalysts. By alloying Ru with Au, the stability of the catalyst was significantly improved. Our present work provided not only an effective OER catalyst that could work in an acidic environment, but also triggered the development of undiscovered solid-solution alloy NPs from immiscible noble metals.

## Conflicts of interest

There are no conflicts to declare.

## Supplementary Material

Supplementary information
