# Supplementary material for: Solid-solution alloy nanoparticles of a combination of immiscible Au and Ru with a large gap of reduction potential and their enhanced oxygen evolution reaction performance
Source: Chem Sci. 2019 Apr 25;10(19):5133–7. doi: 10.1039/c9sc00496c (PMC6524567; doi:10.1039/c9sc00496c)
Supplement: Supplementary file 1 [file SC-010-C9SC00496C-s001.pdf]

Electronic Supplementary Material (ESI) for Chemical Science.
This journal is © The Royal Society of Chemistry 2019

Supporting Information

Solid-solution alloy nanoparticles of a combination of immiscible Au and Ru with a large gap of reduction potential and their enhanced oxygen evolution reaction performance

Quan Zhang, [1] *Kohei Kusada,*[*,1] *Dongshuang Wu,* [1] *Naoki Ogiwara,* [1] *Tomokazu Yamamoto,* [2,3] *Takaaki Toriyama,* [3] *Syo Matsumura,* [2,3,4] *Shogo Kawaguchi,* [5] *Yoshiki Kubota,* [6] *Tetsuo Honma,*[5] *and Hiroshi Kitagawa*[*,1,4]

[1]Division of Chemistry, Graduate School of Science, Kyoto University, Kitashirakawa-Oiwakecho, Sakyo-ku, Kyoto 606-8502, Japan

[2]Department of Applied Quantum Physics and Nuclear Engineering, Kyushu University, 744 Motooka, Nishi-ku, Fukuoka 819-0395, Japan

[3]The Ultramicroscopy Research Center, Kyushu University, Motooka 744, Nishi-ku, Fukuoka 819-0395, Japan

[4]INAMORI Frontier Research Center, Kyushu University, Motooka 744, Nishi-ku, Fukuoka 819-0395, Japan

[5]Japan Synchrotron Radiation Research Insitute (JASRI), SPring-8, 1-1-1 Kouto, Sayo-cho, Sayo-gun, Hyogo 679-5198, Japan

[6]Department of Physical Science, Graduate School of Science, Osaka Prefecture University, 1-1 Gakuen-cho, Naka-ku, Sakai, Osaka 599-8531, Japan

kitagawa@kuchem.kyoto-u.ac.jp; kusada@kuchem.kyoto-u.ac.jp

1. Experimental Procedures

Synthesis of Au_xRu_{1-x} NPs

The Au_xRu_{1-x} alloy NPs were prepared through a polyol reduction method. First, hydrogen tetrabromoaurate (III) hydrate ($HAuBr_4 \cdot nH_2O$, Alfa Aesar) and potassium pentachloronitrosylruthenate (II) ($K_2Ru(NO)Cl_5$, Aldrich) were dissolved into 10 ml diethylene glycol (DEG, Wako) with an appropriate molar ratio (x = 0.1, 0.3, 0.5, 0.7, 0.9. The total amount of the precursors is 0.1 mmol). Then, the metal precursor solution was slowly dropped into 100 ml ethylene glycol (EG, Wako) solution containing 444 mg poly(vinylpyrrolidone) (PVP, 444 mg, MW ≈ 40 000, Wako) at 190 °C. The temperature of the solution was maintained at approximately 190 °C during the dropping process. After that, the solution was kept at 190 °C for another 10 min. Then, the NPs were separated by centrifuging after cooling to room temperature.

Characterizations

Transmission electron microscopy (TEM) images were acquired through a Hitachi HT7700 operated at 100 kV. The high-angle annular dark-field scanning transmission electron microscopy (HAADF-STEM) images and energy-dispersive X-ray spectroscopy (EDX) spectra were obtained on a JEOL JEM-ARM200CF STEM instrument operated at 200 kV. X-ray fluorescence spectroscopy (XRF) was measured using a Rigaku ZSX Primus IV. Synchrotron X-ray diffraction (XRD) patterns were measured at the BL02B2 beamline, SPring-8 at room temperature. The radiation wavelength was 0.58068(1) Å. The Rietveld refinements were performed by using TOPAS3 software developed by Bruker AXS GmbH. X-ray photoelectron spectroscopy (XPS) spectra were obtained by using a Shimadzu ESCA-3400 X-ray photoelectron spectrometer. The Au L_3-edge and Ru K-edge X-ray absorption near edge structure

(**XANES**) were collected in the transmission mode under ambient conditions using a Si (311) double-crystal monochromator at the BL14B2 beamline, SPring-8. The samples are finely ground in powder, mixed with a boron nitride powder in a suitable ratio and pressed into pellets for XANES measurement.

Electrochemical Measurements

Electrochemical characterization was conducted by using a standard three-electrode system. A Pt wire and an Ag/AgCl (3.5 M NaCl) electrode were used as the counter and reference electrodes, respectively. A CHI 760E electrochemical analyzer (CH Instruments) was used for recording the electrochemical data. To measure the catalytic properties, the Au_xRu_{1-x} solids-solution NPs were first loaded on the carbon black (VXC 72R) with a 20 wt% of metal. Here, the PVP amounts of the Au_xRu_{1-x} (x = 0, 0.1, 0.3, 0.5, 0.7, 0.9, and 1.0) NPs were determined from the elemental analysis to be 14.5 wt%, 13.7 wt%, 7.2 wt%, 5.7 wt%, 7.9 wt%, 8.5 wt% and 17.3 wt%, respectively. Then, 10 mg catalysts were added to 1 ml mixed solution (300 μl water, 600 μl isopropanol and 100 μl Nafion solution (Sigma Aldrich, 5 wt%)) followed by sonication for 60 min to form a homogeneous ink. The catalyst inks were uniformly cast onto a glassy carbon electrode (5 mm-diameter with a surface area of 0.196 cm^2) with a 0.2 mg cm^{-2} loading for all samples, which was used as a working electrode. All the electrodes were purchased from ALS Co., Ltd. The working electrode surface was polished with 1 μM diamond and 0.05 μM alumina solutions (ALS Co., Ltd.) before loading the catalysts. Before OER catalytic activity testing, the H_2SO_4 solutions were purged by Ar for 30 min. Ar was kept flowing above the solution to maintain an inert atmosphere during the measurement. The OER polarization curves were recorded over the potential range +0.95 to +1.5 V (vs. Ag/AgCl) at a scan rate of 5 mV / s. The

working electrode was continuously rotating at 1600 rpm during the measurements. Chronopotentiometric measurements for $Au_{0.3}Ru_{0.7}$ and Ru were carried out under a constant current density of 2.5 mA / cm^2 for 1 h. For all measurements, the current density was *iR*-corrected using the measured solution resistance. The current density was finally normalized by the surface area of the working electrode.

2. Reduction Potentials of Noble Metals

Table S1. Reduction Potentials of the Noble Metals Under Standard Conditions (25 °C, 1 atm). SHE: Standard Hydrogen Electrode [1]

Reduction reaction	E^o (V vs SHE)
$Ru^{3+} + 3e^- \rightarrow Ru$	0.455
$Rh^{3+} + 3e^- \rightarrow Rh$	0.758
$Pd^{2+} + 2e^- \rightarrow Pd$	0.951
$Ag^+ + e^- \rightarrow Ag$	0.800
$Ir^{3+} + 3e^- \rightarrow Ir$	1.156
$Pt^{2+} + 2e^- \rightarrow Pt$	1.188
$Au^{3+} + 3e^- \rightarrow Au$	1.498

3. Au–Ru Phase Diagram

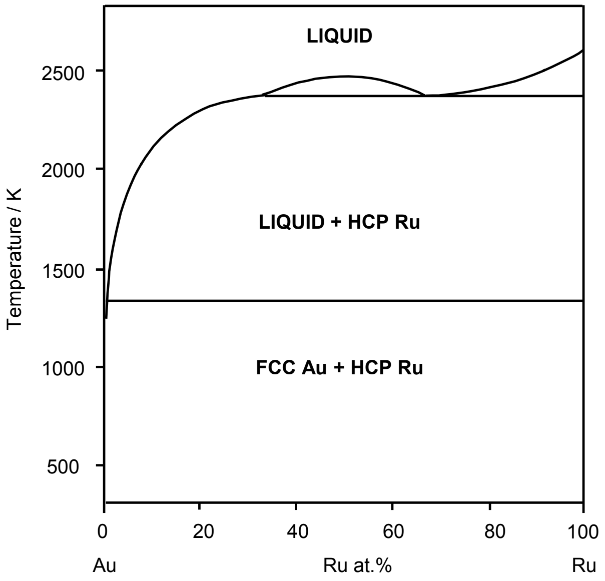

Figure S1. Phase diagram of Au–Ru system [2]

4. TEM Images of Au_xRu_{1-x} NPs

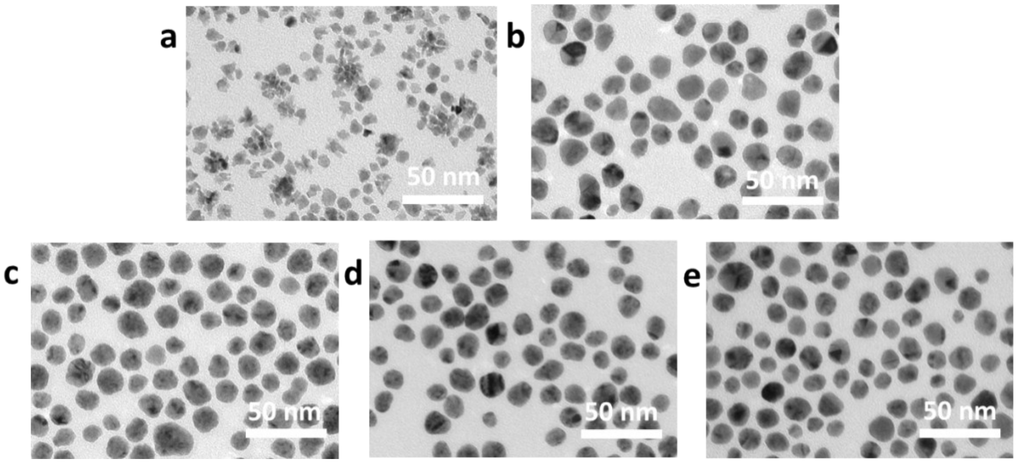

Figure S2. TEM images of the Au_xRu_{1-x} NPs. (a)-(e) are the TEM images of $Au_{0.1}Ru_{0.9}$, $Au_{0.3}Ru_{0.7}$, $Au_{0.5}Ru_{0.5}$, $Au_{0.7}Ru_{0.3}$ and $Au_{0.9}Ru_{0.1}$ NPs respectively.

5. Metal Compositions of Au_xRu_{1-x} alloy NPs

Table S2. Metal Compositions of Au_xRu_{1-x} alloy NPs Determined from EDX and XRF Analysis

Sample	$Au_{0.1}Ru_{0.9}$	$Au_{0.3}Ru_{0.7}$	$Au_{0.5}Ru_{0.5}$	$Au_{0.7}Ru_{0.3}$	$Au_{0.9}Ru_{0.1}$
Au : Ru (EDX)	0.13 : 0.87	0.31 : 0.69	0.51 : 0.49	0.72 : 0.28	0.92 : 0.08
Au : Ru (XRF)	0.15 : 0.85	0.32 : 0.68	0.53 : 0.47	0.74 : 0.26	0.93 : 0.07

6. STEM−EDX Maps of Au_xRu_{1-x} NPs

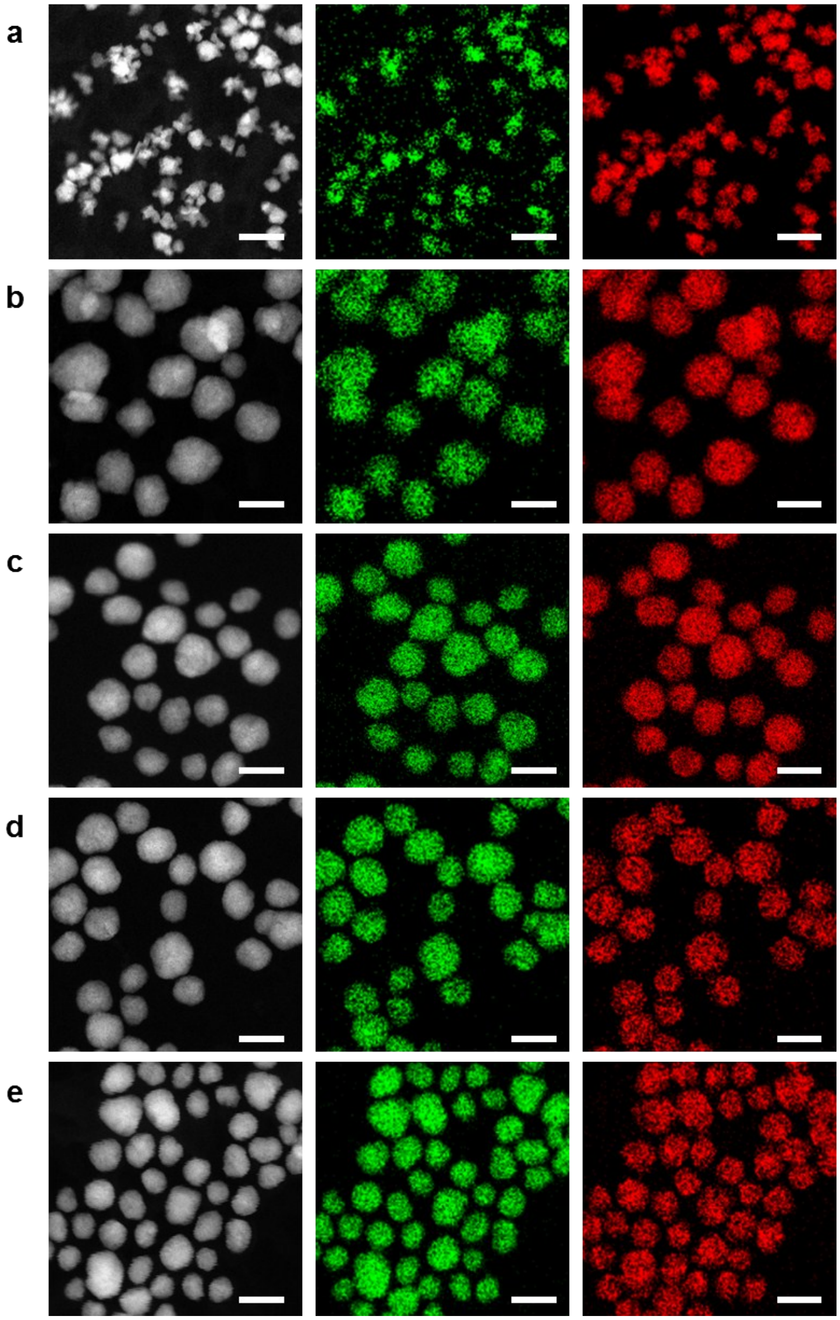

Figure S3. HAADF-STEM Images and the corresponding Au-*L* (green) and Ru-*L* (red) STEM−EDX maps of (a) $Au_{0.1}Ru_{0.9}$, (b) $Au_{0.3}Ru_{0.7}$, (c) $Au_{0.5}Ru_{0.5}$, (d) $Au_{0.7}Ru_{0.3}$ and (e) $Au_{0.9}Ru_{0.1}$ NPs. The scale bars shown in (a)-(e) are 20 nm.

7. Rietveld Refinement of the XRD Patterns for Au_xRu_{1-x} NPs

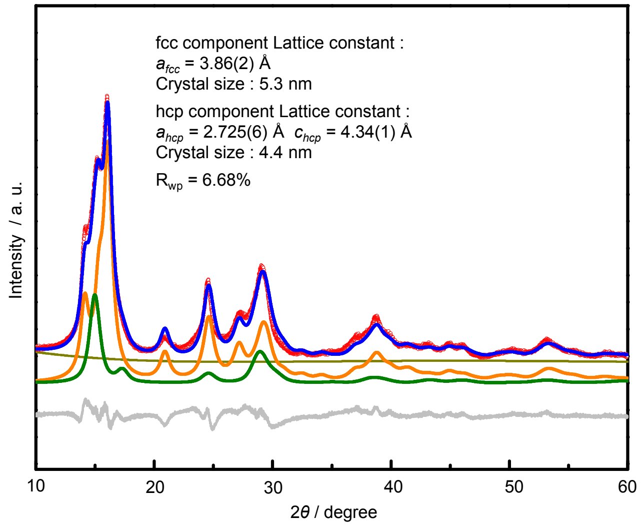

Figure S4. Rietveld refinement for $Au_{0.1}Ru_{0.9}$ NPs. The diffraction patterns are shown as red circles. The calculated patterns are shown as the blue line. The difference profile, the background profile, the fitting curves of the fcc components, and the fitting curves of the hcp components are shown as gray, dark yellow, green, and orange lines, respectively.

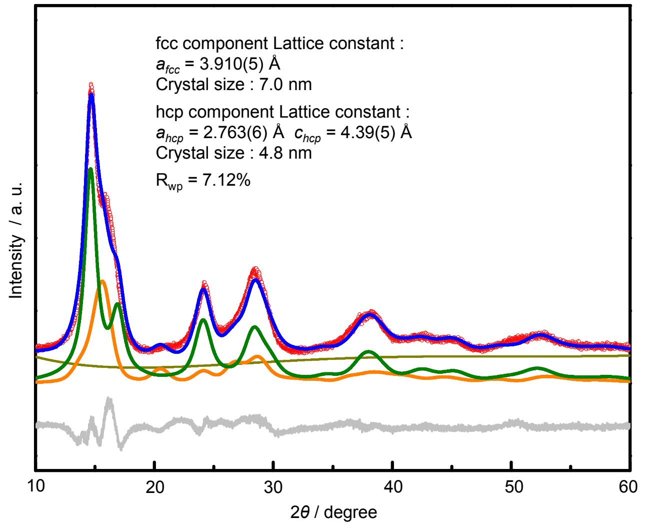

Figure S5. Rietveld refinement for $Au_{0.3}Ru_{0.7}$ NPs. The diffraction patterns are shown as red circles. The calculated patterns are shown as the blue line. The difference profile, the background profile, the fitting curves of the fcc components, and the fitting curves of the hcp components are shown as gray, dark yellow, green, and orange lines, respectively.

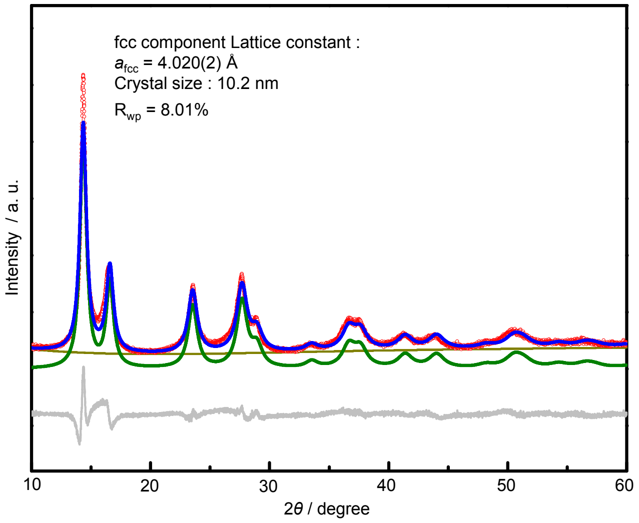

Figure S6. Rietveld refinement for $Au_{0.7}Ru_{0.3}$ NPs. The diffraction patterns are shown as red circles. The calculated patterns are shown as the blue line. The difference profile, the background profile, and the fitting curves of the fcc components are shown as gray, dark yellow, and green, respectively.

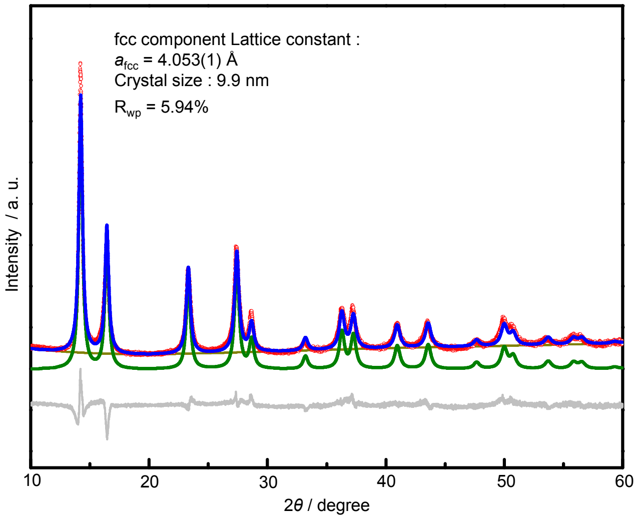

Figure S7. Rietveld refinement for $Au_{0.9}Ru_{0.1}$ NPs. The diffraction patterns are shown as red circles. The calculated patterns are shown as the blue line. The difference profile, the background profile, and the fitting curves of the fcc components are shown as gray, dark yellow and green lines, respectively.

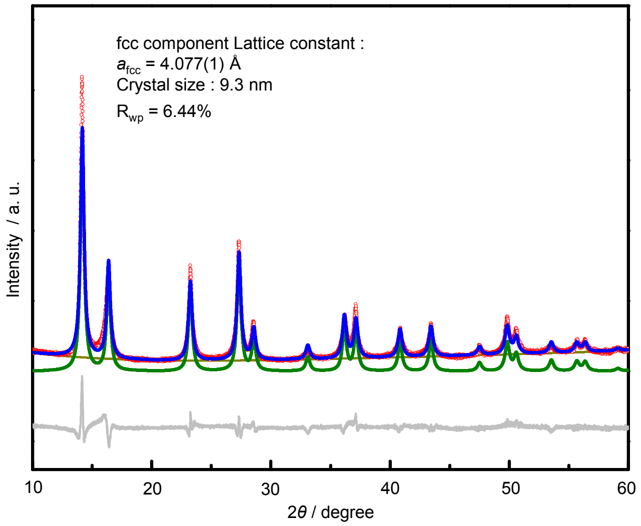

Figure S8. Rietveld refinement for Au NPs. The diffraction patterns are shown as red circles. The calculated patterns are shown as a blue line. The difference profile, the background profile, and the fitting curves of the fcc components are shown as gray, dark yellow and green lines, respectively.

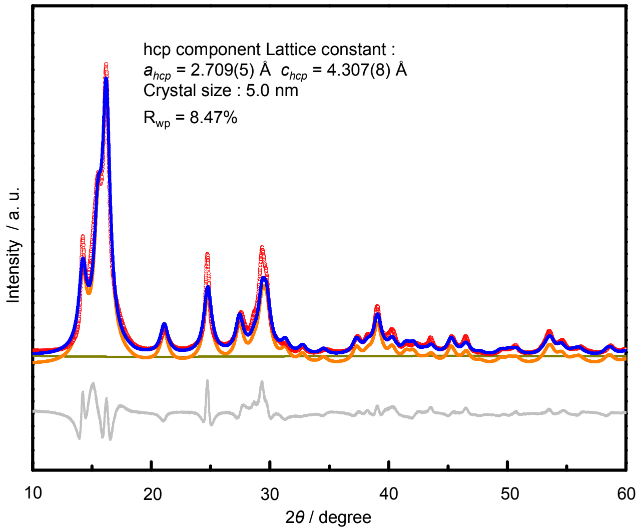

Figure S9. Rietveld refinement for Ru NPs. The diffraction patterns are shown as red circles. The calculated patterns are shown as blue line. The difference profile, the background profile, and the fitting curves of the hcp components are shown as gray, dark yellow and orange lines, respectively.

8. TEM Images of the Au_xRu_{1-x}/C Catalysts

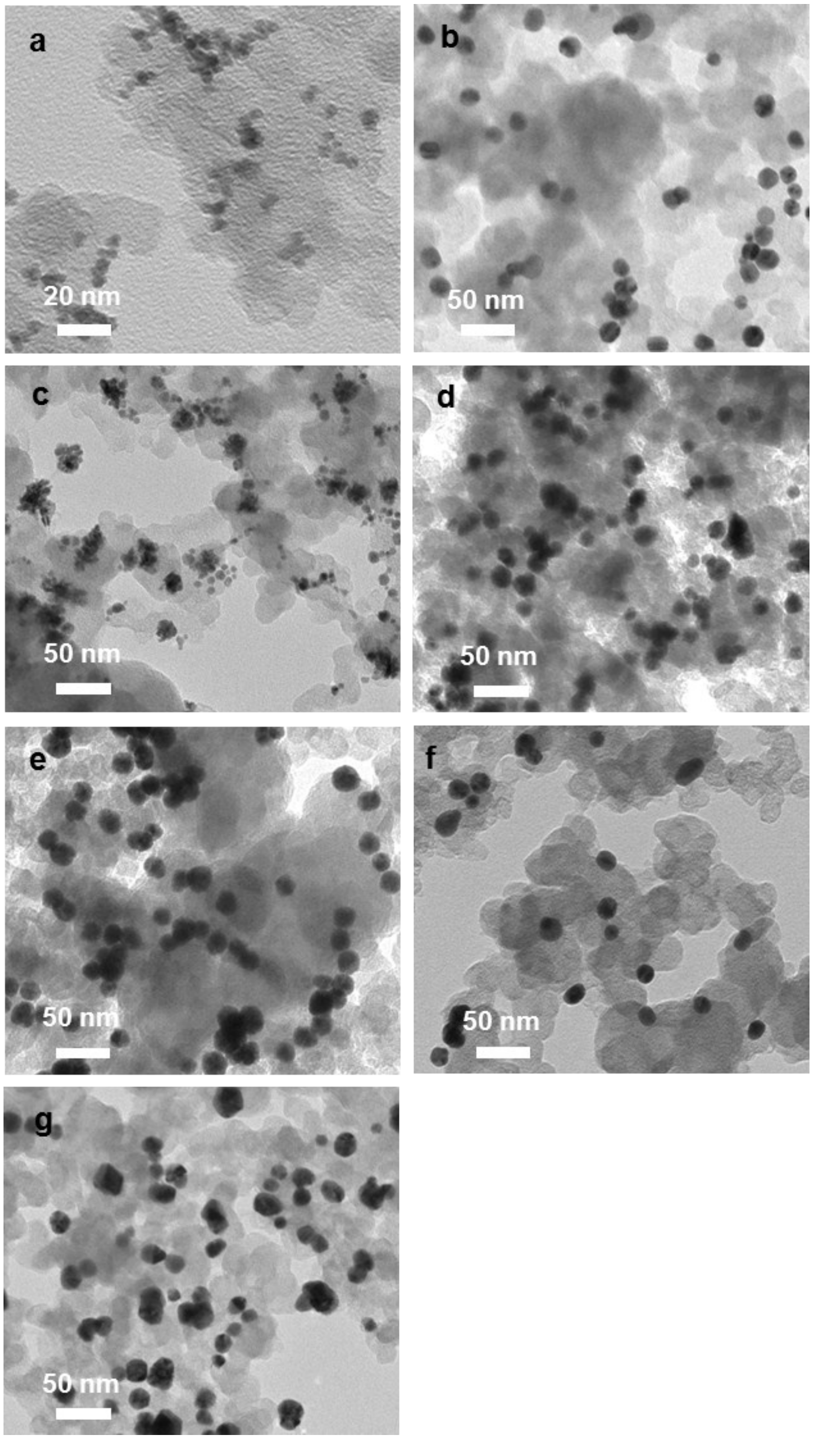

Figure S10. TEM images of the Au_xRu_{1-x}/C catalysts. (a)–(g) are the TEM images of Ru, Au, $Au_{0.1}Ru_{0.9}$, $Au_{0.3}Ru_{0.7}$, $Au_{0.5}Ru_{0.5}$, $Au_{0.7}Ru_{0.3}$ and $Au_{0.9}Ru_{0.1}$ NPs loading on the carbon black respectively.

9. Chronopotentiometric Stability of Ru and $Au_{0.1}Ru_{0.9}$ Catalysts

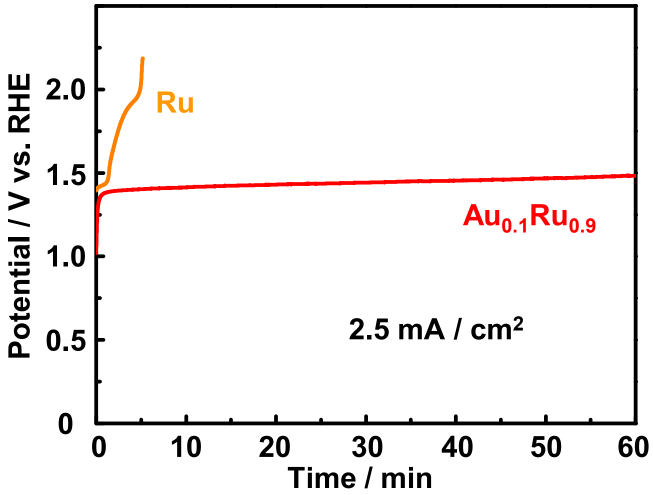

Figure S11. Chronopotentiometry curves of $Au_{0.1}Ru_{0.9}$ catalysts compared to Ru catalyst under constant current density of 2.5 mA/cm^2 for 1 h.

10. Structure of the Catalysts after the Chronopotentiometric Stability Measurement

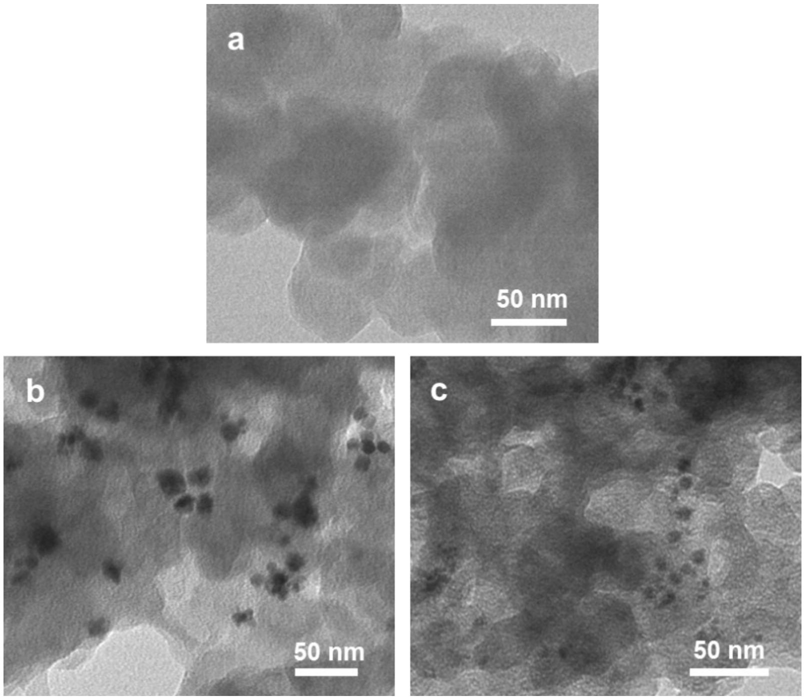

Figure S12. TEM images of Ru/C (a), $Au_{0.3}Ru_{0.7}$/C (b) and $Au_{0.1}Ru_{0.9}$/C (c) catalysts after stability test.

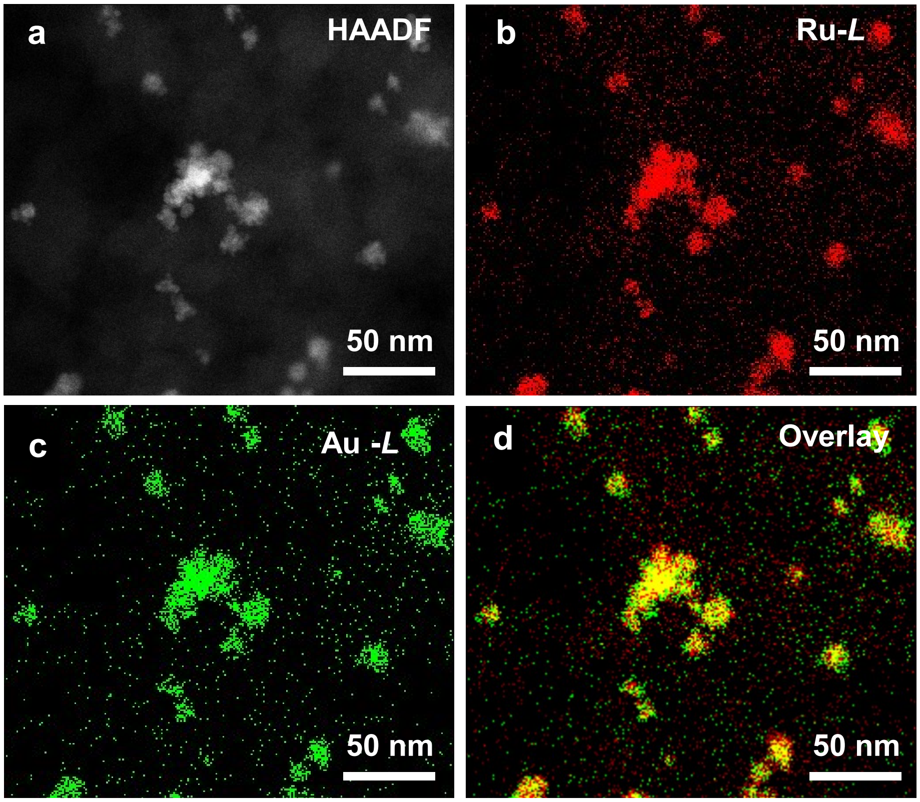

Figure S13. STEM images and the EDX maps of the $Au_{0.3}Ru_{0.7}$ catalysts after the chronopotentiometric stability measurement in low magnification. (a) HAADF- STEM image, (b)−(d) the corresponding Ru-*L* (red), Au-*L* (green) and overlay STEM−EDX maps of NPs in (a).

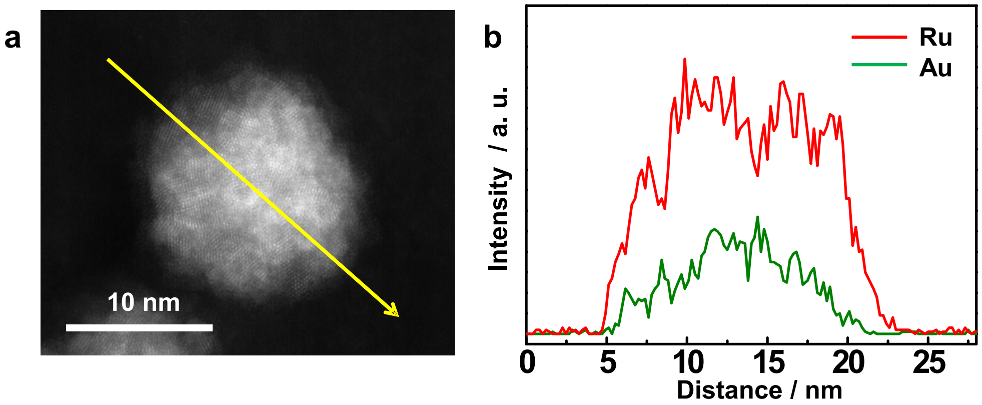

Figure S14. (a) HAADF- STEM image of the $Au_{0.3}Ru_{0.7}$ catalysts after the chronopotentiometric stability measurement in low magnification, (b) the EDX line profiles of the NP along the arrow shown in (a).

11. XPS Analysis of Au_xRu_{1-x} alloy NPs

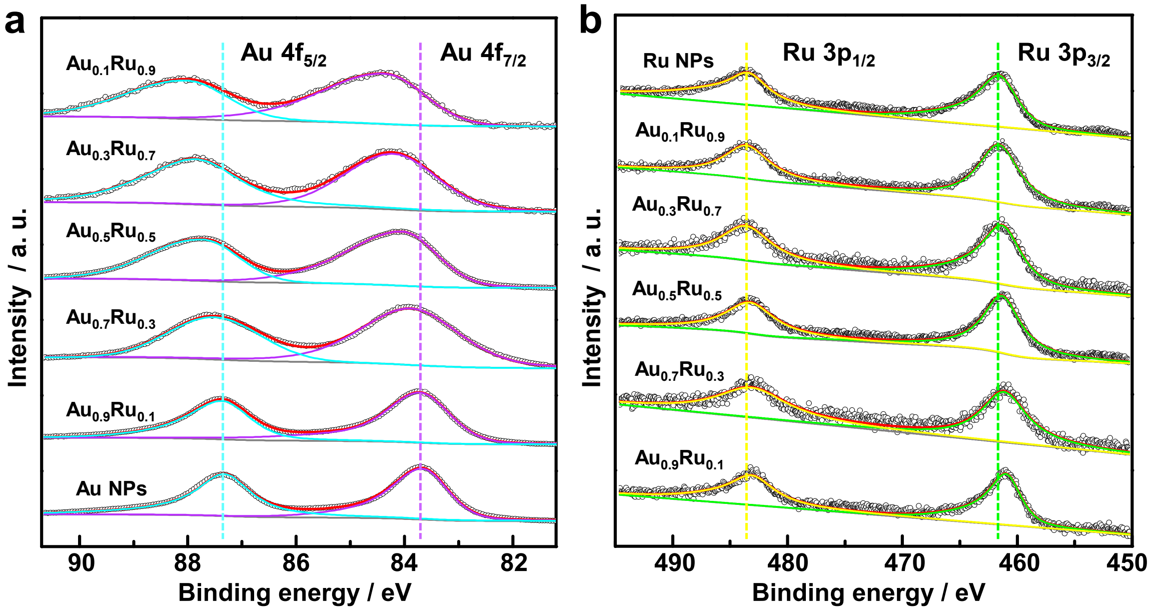

Figure S15. (a) Au 4*f* and (b) Ru 3*p* core-level XPS spectra of Au_xRu_{1-x}, Au and Ru NPs.

Table S3. Binding energies of Au 4*f* and Ru 3*p*

Sample	Binding energy (ev)			
	**Au 4$f_{5/2}$**	**Au 4$f_{7/2}$**	**Ru 3$p_{1/2}$**	**Ru 3$p_{3/2}$**
**Ru NPs**			483.80	461.63
**$Au_{0.1}Ru_{0.9}$**	88.05	84.38	483.69	461.52
**$Au_{0.3}Ru_{0.7}$**	87.89	84.22	483.57	461.38
**$Au_{0.5}Ru_{0.5}$**	87.71	84.04	483.43	461.25
**$Au_{0.7}Ru_{0.3}$**	87.53	83.86	483.33	461.08
**$Au_{0.9}Ru_{0.1}$**	87.40	83.73	483.11	460.94
**Au NPs**	87.33	83.65		

12. Reaction Pathway of OER in Acid Solution

Table S4. Overall Reaction Pathway of OER in Acid Solution [3]

**Overall reaction:**	**Reaction pathway:**
$2H_2O \rightarrow O_2 + 4H^+ + 4e^-$	$* + H_2O \rightarrow *OH + H^+ + e^-$
	$*OH \rightarrow *O + H^+ + e^-$
	$*O + H_2O \rightarrow *OOH + H^+ + e^-$
	$*OOH \rightarrow *O_2 + H^+ + e^-$
	$*O_2 \rightarrow * + O_2$

The * represents active site on the metal surface.

References

[1] P. Vanýsek, in *CRC Handbook of Chemistry and Physics, 83rd Edition*, (Eds.: D. R. Lide), Taylor & Francis, Boca Raton, Florida, USA **2002**.

[2] H. Okamoto, T. B. Massalski, *J. Phase Equilib.* **1984**, *5*, 388.

[3] J. Rossmeisl, A. Logadottir, J. K. Nørskov, *Chem. Phy.* **2005**, *319*, 178.
